# Comparison of the effects between MPL and JAK2V617F on thrombosis and peripheral blood cell counts in patients with essential thrombocythemia: a meta-analysis

**DOI:** 10.1007/s00277-021-04617-6

**Published:** 2021-08-12

**Authors:** Erpeng Yang, Mingjing Wang, Ziqing Wang, Yujin Li, Xueying Wang, Jing Ming, Haiyan Xiao, Richeng Quan, Weiyi Liu, Xiaomei Hu

**Affiliations:** 1 Department of Haematology, Xiyuan Hospital of China Academy of Chinese Medical Sciences, No.1 Xiyuan Caochang Road, Haidian District, Beijing, 100091 China; 2 Graduate School of China Academy of Chinese Medical Sciences, Beijing, 100700 China; 3 Xiyuan Clinical Medical College of Beijing University of Traditional Chinese Medicine, Beijing, 100029 China

**Keywords:** MPL, JAK2V617F, Thrombosis, Blood cells, Essential thrombocythemia, Meta-analysis

## Abstract

To assess the effects between MPL and JAK2V617F on the thrombosis risk and peripheral blood cell counts in patients with essential thrombocythemia (ET), we identified eligible studies from PubMed, Embase, and the Cochrane Library. Seven studies were ultimately included in this meta-analysis. All studies reported the peripheral blood cell counts of ET patients, and three of them reported the eligible thrombotic events. In comparing the effect of MPL versus JAK2V617F on thrombosis, 1257 ET patients (73 MPL + and 1184 JAK2V617F +) were included. MPL-positive (MPL +) ET patients had a higher risk of thrombosis than JAK2V617F-positive (JAK2V617F +) ET patients [RR = 1.80 (1.08–3.01), *P* = 0.025]. And 3453 ET patients (138 MPL + and 3315 JAK2V617F +) were included in the comparison of peripheral blood cell counts. Platelet counts of MPL + ET patients were higher than that of JAK2V617F + ET patients [WMD = 81.18 (31.77–130.60), *P* = 0.001]. MPL + ET patients had lower hemoglobin [WMD =  − 11.66 (− 14.32 to − 9.00), *P* = 0.000] and white blood cell counts [WMD =  − 1.01 (− 1.47 to − 0.56), *P* = 0.000] than JAK2V617F + ET patients. These findings indicate that the MPL mutation is a high-risk factor for thrombosis in ET patients, and it may be rational to include MPL mutation in the revised IPSET as a criterion for thrombosis prediction scores. And given the differences in peripheral blood, it is necessary to further study whether MPL + ET patients differ from JAK2V617F + ET patients in bleeding and survival.

## Introduction

Essential thrombocythemia (ET) is a kind of myeloproliferative neoplasms (MPN), characterized by the proliferation of bone marrow megakaryocytes and the increase of peripheral blood platelet counts. The median overall survival (OS) time for patients with ET is 18 years [1]. The main treatment of ET is to prevent thrombosis, and the incidence of thrombosis in ET is 21% after diagnosis [1–3]. As the three driver genes of ET, the mutation rates of JAK2V617F, MPL, and CALR are about 60%, 3%, and 20% respectively [4].

The risk of thrombosis and peripheral blood cell counts have important clinical significance to the treatment and prognosis of ET patients. Compared with JAK2V617F + patients, CALR + patients and the triple negative (TN) patients have a lower risk of thrombosis [5–8]. Because of the low frequency of MPL mutation in ET, the risk of thrombosis and the peripheral blood cell counts of MPL + patients have not been clearly defined. It had previously been reported that MPL mutations had a similar effect on thrombotic events compared with JAK2V617F mutations [9]. However, MPL mutations had also been shown to be more likely to promote thrombosis than JAK2V617F mutations in patients with ET [10]. These two studies involved a small number of patients, and the results were obviously controversial. Hence, we performed this meta-analysis to compare thrombotic events and peripheral blood cell counts between MPL + and JAK2V617F + ET patients.

## Methods

### Protocol registration

The protocol for this review was registered in advance in the International Prospective Register of Systematic Reviews (PROSPERO registration number, CRD42021241097) [11].

### Literature search and search strategy

We conducted a systematic literature search on PubMed, EMBASE, and the Cochrane Library for potentially relevant studies published from inception to December 31, 2020. The search terms were as follows: “JAK2V617F,” “MPL,” “essential,” “thrombocytosis,” “thrombocythemia,” and “thrombosis.”

### Selection criteria

Only papers meeting all of the following criteria were included: (1) They were published as original articles from inception to 31 December 2020. (2) They diagnosed patients with ET according to criteria of the Polycythemia Vera Study Group (PVSG) or the World Health Organization (WHO) criteria [12–14]. (3) The number of MPL + or JAK2V617F + ET patients must all be greater than or equal to 5. (4) They provided data on thrombotic events after diagnosis or peripheral blood cell counts (hemoglobin, white blood cells, and platelets) at diagnosis/enrollment. (5) Multiple reports of a study were considered as one publication and only the most complete article was examined. (6) Review articles, case reports, and conference abstracts were excluded. Two reviewers independently screened the database and identified eligible studies. Disagreements were resolved through discussion.

### Data extraction

Two reviewers (Erpeng Yang and Mingjing Wang) independently reviewed all articles that met the inclusion criteria. The following information was extracted and listed for each eligible study: first author, year, study location, gender, number of people, diagnostic criteria, peripheral blood cell counts, and data on thrombotic events. In this study, thrombotic events after diagnosis and peripheral blood cell counts at diagnosis/enrollment were selected as the results. If the original study only provided the median and range of the patient’s peripheral blood cell counts, the meta-analysis could not be completed directly. We would first contact the author and ask whether we could get the raw data. When we finally got no response, we would estimate the mean value and standard deviation by using the median, range, and sample size [15].

### Quality assessment

Two authors (Erpeng Yang and Mingjing Wang) independently assessed the methodological quality of each study by using the Newcastle–Ottawa Quality Assessment Scale (NOS) for cohort studies [16]. Another reviewer (Ziqing Wang) addressed any discrepancies. There are nine items on the scale, which are divided into three categories: selection (four items), comparability (two items), and cohort design outcomes (three items). According to the NOS, the quality of these studies was divided into three types: high (7–9 points), medium (4–6 points), and low (1–3 points).

### Statistical analysis

Stata version14.0 software (Stata Corp., College Station, TX, USA) was used to calculate the effects of MPL and JAK2V617F mutations on thrombosis and peripheral blood cell counts of ET patients. The number of patients and thrombotic events in each group were used to calculate the relative risks (RRs) and the 95% confidence intervals [17, 18]. The number of patients and the peripheral blood counts were used to calculate the weighted mean differences (WMDs) and the 95% confidence intervals [19]. We assessed the statistical heterogeneity by *Q*-test and *I*^2^ statistics. When *I*^2^ was > 50% or *P* < 0.10, the random-effects model results would be used; otherwise, the fixed-effects model results were preferred. Funnel plots, Begg’s test, and Egger’s test were used to detect publication bias if the final number of included studies was not less than 10 [20–22]. An asymmetric funnel plot or a *P* value of less than 0.05 for either of the two tests was considered as publication bias. For all the outcomes, a *P* value of less than 0.05 was considered statistically significant.

## Results

### Search results

**Fig. 1:**
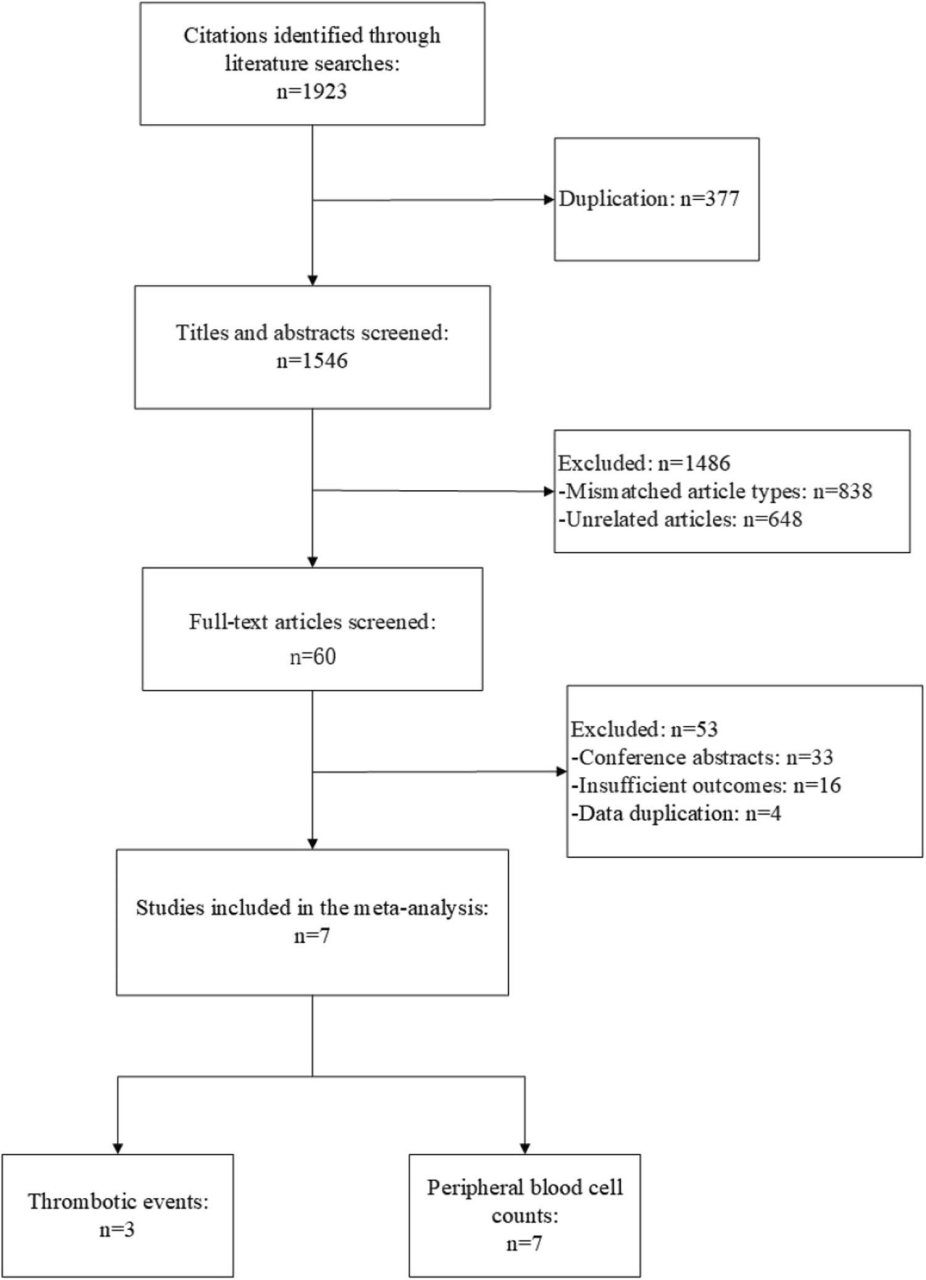
Flow diagram of study selection

A total of 1923 studies were obtained from the initial search, of which 377 were excluded because of duplication. After screening the titles and abstracts of 1546 studies, 60 studies were left for full-text review. Based on the selection criteria, 7 studies were eventually included. The study selection process is shown in Fig. 1.

### Characteristics of included studies

The meta-analysis included 5 prospective studies [10, 23–26] and 2 retrospective studies [27, 28], including a total of 3453 patients with ET (138 MPL + and 3315 JAK2V617F +). There were 3 studies [10, 23, 25] that included eligible data on thrombotic events after diagnosis. Peripheral blood cell counts were reported in all studies. In these studies, four were from Europe [10, 23, 26, 28], two from China [24, 27], and one from the USA [25]. The characteristics of these studies are reported in Table 1.

**Table 1:** Baseline patient characteristics of the included studies

Study ID	Region	Diagnostic criteria	Mutation type	No	Thrombosis	Median age, years (range)	Sex (M/F)	Hemoglobin, g/L*	Leucocyte count, × 10^9^/L*	Platelet count, × 10^9^/L*
Vannucchi 2008 [10]	Italy	PVSG 1997 /WHO 2001	MPL JAK2V617F	30 546	6 32	56 (22–84) 56 (15–97)	9/21 170/376	134 ± 13 142 ± 21	8.8 ± 3.1 9.5 ± 3.1	956 ± 331 791 ± 211
Beer 2008 [23]	UK and NI, Australia, France, New Zealand	PVSG 1997	MPL JAK2V617F	32 411	4 36	67 (48–77) 60 (39–77)	15/17 156/255	133 ± 12 145 ± 14	9.9 ± 2.4 10.6 ± 3.4	1040 ± 272 900 ± 274
Fu 2014 [24]	China	WHO 2008	MPL JAK2V617F	6 240	N/A N/A	50 (32–61) 56 (21–85)	3/3 99/141	147 ± 85 144 ± 17	11.4 ± 7.8 11.1 ± 3.9	1690 ± 1273 734 ± 327
Tefferi 2014 [25]	USA, Italy	WHO 2001/2008	MPL JAK2V617F	11 227	3 55	64 (23–85) 58 (14–88)	5/6 84/143	127 ± 76 141 ± 14	8.9 ± 6.5 9.8 ± 8.8	1157 ± 834 862 ± 425
Li 2017 [27]	China	WHO 2008	MPL JAK2V617F	9 819	N/A N/A	58 (22–71) 62 (15–95)	6/3 386/433	136 ± 79 138 ± 25	8.8 ± 5.5 12.2 ± 12.4	904 ± 577 800 ± 589
Alvarez-Larrán 2020 [26]	Spain	WHO after 2000	MPL JAK2V617F	45 960	N/A N/A	65 (10–89) 64 (17–95)	11/34 369/591	131 ± 17 145 ± 16	7.7 ± 2.0 8.9 ± 3.3	688 ± 224 668 ± 350
Prejzner 2020 [28]	Poland	WHO 2008	MPL JAK2V617F	5 112	N/A N/A	66 (54–78) 59 (23–88)	2/3 38/74	128 ± 12 145 ± 15	9.2 ± 5.5 10.4 ± 6.3	941 ± 233 934 ± 932

Abbreviations: *F* female, M male, *N/A* not applicable, *PVSG* Polycythemia Vera Study Group, *WHO* World Health Organization

^*^Mean value ± SD

### Quality assessment of included studies

The median overall score of NOS outcomes of the included studies was 8 (range 7–9), which indicated that the methodological quality was high (Table 2).

**Table 2:** Quality assessment of NOS

Study ID	Selection				Comparability	Outcome			Score
	Representativeness of exposed cohort	Selection of non-exposed cohort	Ascertainment of exposure	Outcome not present at beginning of study		Assessment of outcome	Follow-up length	Follow-up adequacy	
Vannucchi 2008 [10]	★	★	★	★	★	★	★	★	8
Beer 2008 [23]	★	★	★	★	★★	★	★	★	9
Fu 2014 [24]	★	★	★	★	★	★	★	★	8
Tefferi 2014 [25]	★	★	★	★	★★	★	★	★	9
Li 2014 [27]	★	★	★	-	★	★	★	★	7
Alvarez-Larrán 2020 [26]	★	★	★	★	★	★	★	★	8
Prejzner 2020 [28]	★	★	★	-	★	★	★	★	7

Note: A study can be awarded a maximum of one star for each numbered item within the Selection and Outcome categories. A maximum of two stars can be given for Comparability

### Outcomes of thrombotic events

**Fig. 2:**
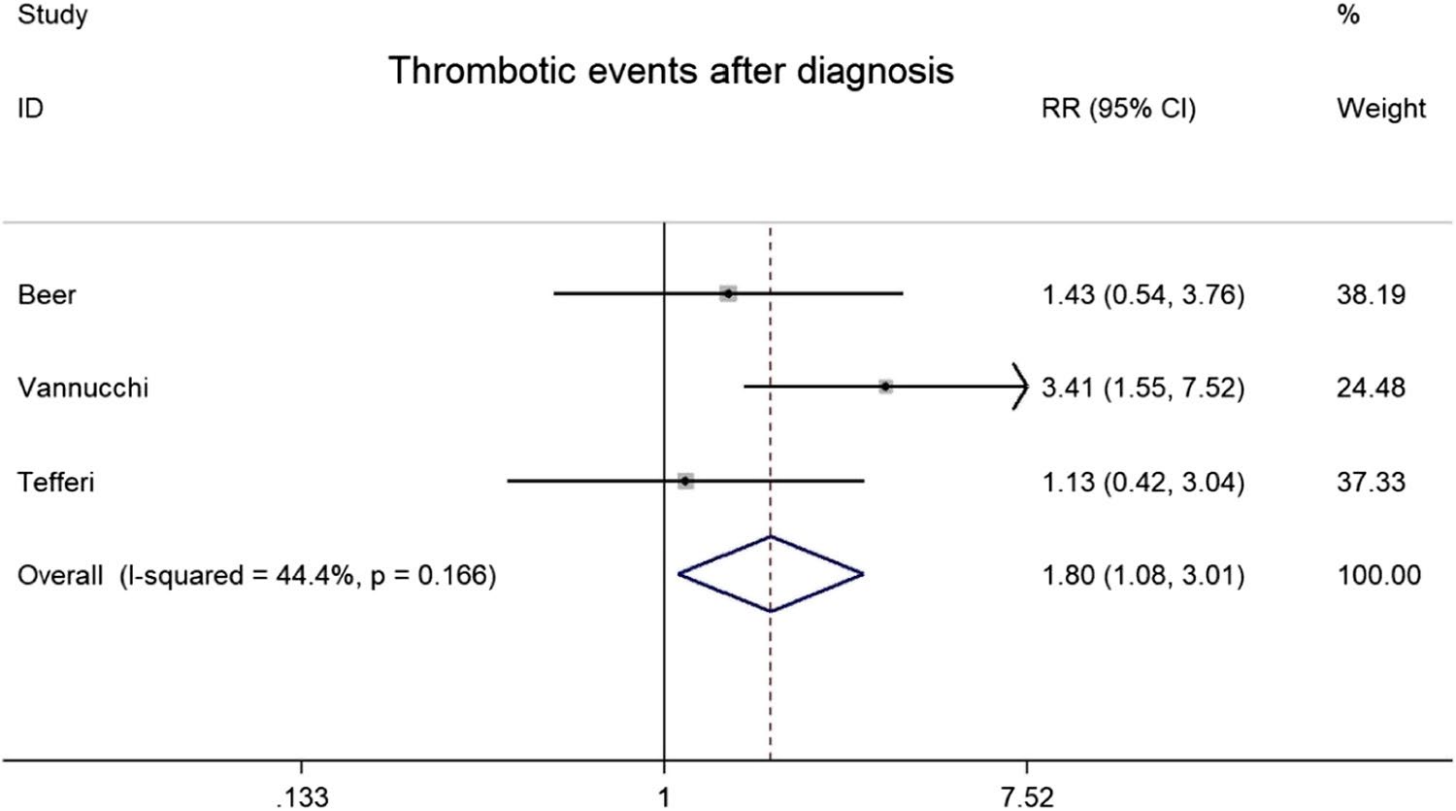
Forest plots of the RRs for the risk of thrombosis between MPL + and JAK2V617F + ET patients

As shown in Fig. 2, three studies with 1257 ET patients (73 MPL + and 1184 JAK2V617F +) reported data on thrombosis. Heterogeneity was not statistically significant [*I*^2^ = 44.4%, *P* = 0.166]. RRs were combined using the Mantel–Haenszel fixed effects model to estimate pooled point estimates and their confidence intervals [18]. In this population, MPL + patients had a higher risk of thrombosis than JAK2V617F + patients [RR = 1.80 (1.08–3.01), *P* = 0.025].

### Outcomes of peripheral blood cell counts

**Fig. 3:**
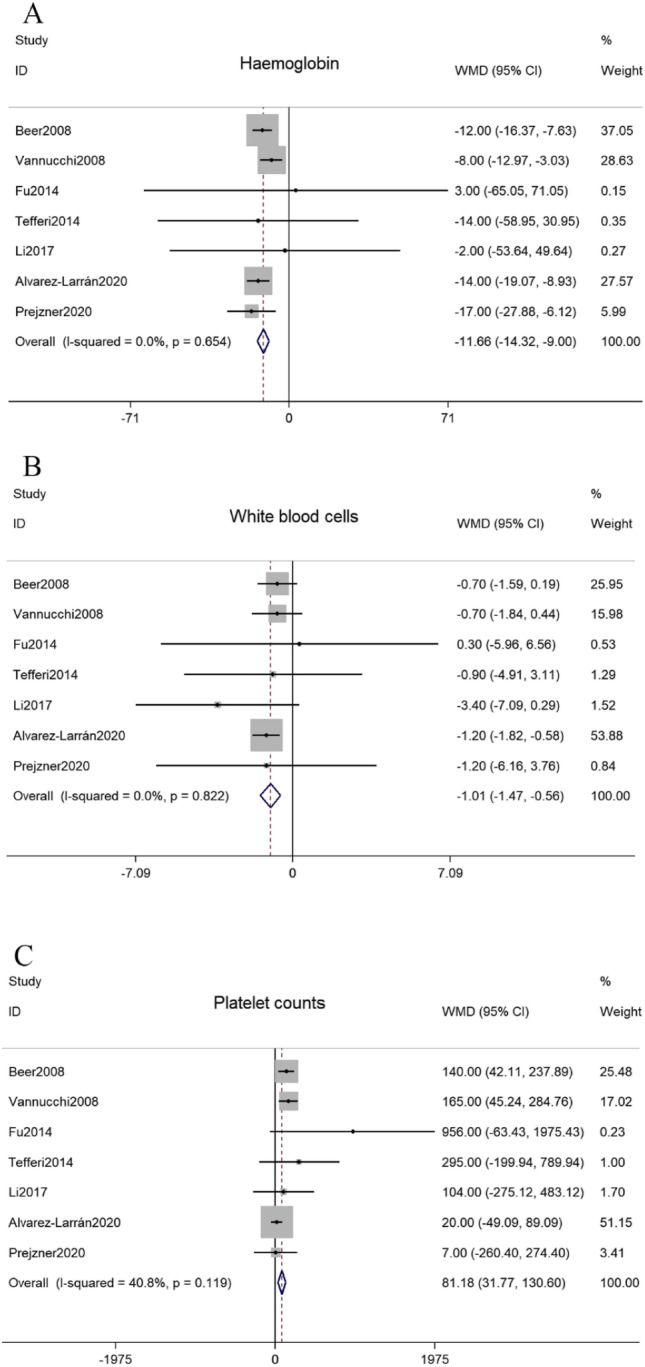
Forest plots of the WMDs for the peripheral blood cell counts between MPL + and JAK2V617F + ET patients. **A** for the haemoglobin levels. **B** for the white blood cell counts. **C** for the platelet counts

As shown in Fig. 3A–C, data for peripheral blood cell counts were obtained from 7 studies. There were 3453 ET patients, including 138 MPL + and 3315 JAK2V617F + patients. In this population, MPL + ET patients had lower hemoglobin levels than JAK2V617F + ET patients [WMD =  − 11.66 (− 14.32 to − 9.00), *P* = 0.000] and the heterogeneity between these studies was not important [*I*^2^ = 0, *P* = 0.654] [29]. MPL + patients had lower white blood cell counts than JAK2V617F + patients [WMD =  − 1.01 (− 1.47 to − 0.56), *P* = 0.000] and the heterogeneity between studies was not important [*I*^2^ = 0, *P* = 0.822] [29]. In addition, platelet counts were higher in patients with MPL mutation compared to those with JAK2V617F mutation [WMD = 81.18 (31.77–130.60), *P* = 0.001], and there was not statistically significant heterogeneity between studies [*I*^2^ = 40.8%, *P* = 0.119].

## Discussion

Thrombotic events severely affect the quality of life and longevity of ET patients. JAK2V617F mutation is an established risk factor for thrombosis [30–33]. However, due to the low mutation rate of MPL in ET patients, there are few reports about the thrombotic events. Many thrombotic events had been reported without in-depth follow-up, so there was little mention of thrombotic events after diagnosis in patients with ET [26, 34–36]. The thrombotic events that occurred at the time of diagnosis and after are clearly more relevant to the disease than the thrombotic events prior to the diagnosis of ET. Because the time between actual onset and diagnosis is not clear, thrombotic events of ET patients at diagnosis are less comparable than those after diagnosis. In contrast, patients have a definite follow-up period after diagnosis, so their thrombotic event results can better reflect the actual risk of thrombosis. This meta provided a better understanding of the thrombotic risk and clinical blood cells profiles of MPL + ET patients by comparing them to JAK2V617F + ET patients.

The seven papers included in this research were of high quality. JAK2V617F mutation is an established risk factor for thrombosis [30–33], which has been included in the International Prognostic Score of thrombosis in World Health Organization-essential thrombocythemia (IPSET-thrombosis) [37] and the later revised IPSET [38, 39]. This result of this study showed that the MPL mutation promoted thrombosis more than the JAK2V617F mutation. Therefore, it may be rational to include MPL mutation in the revised IPSET as a criterion for thrombosis prediction scores. In this study, platelet counts of MPL + patients were higher than that of JAK2V617F + patients, while white blood cell counts and hemoglobin were lower than in JAK2V617F + patients, which was consistent with Beer’s study [23]. In ET patients, platelet count is closely related to bleeding and other complications, and white blood cell count is closely related to survival prognosis [4, 40]. Based on the above correlations, it is necessary to further compare the bleeding and survival between MPL + patients and JAK2 + ET patients. It should be noted that some instances of MPL-mutated ET might actually represent prefibrotic primary myelofibrosis (pre-PMF) [41]. There were no statistically significant differences in hemoglobin levels, white blood cell counts, platelet counts, and post-diagnostic thrombosis between MPL + ET patients and those reclassified as pre-PMF from MPL + ET [41]. Therefore, this factor had little impact on the results of this study.

The following are the main limitations of our meta-analysis. The number of included studies was relatively small and the publication bias could not be analyzed. In addition, these studies involving thrombosis were limited to Western populations and lacked patients from multiple regions. In view of the limited number of studies included in the analysis, our meta-analysis results should be confirmed in future studies comparing the effects of MPL mutation with JAK2V617F mutation on thrombosis in ET patients.

## Conclusion

In conclusion, the MPL mutation is a high-risk factor for thrombosis in ET patients, and it may be rational to include MPL mutation in the revised IPSET as a criterion for thrombosis prediction scores. And given the differences in peripheral blood, we recommend further studies to determine whether MPL + ET patients differ from JAK2V617F + ET patients in bleeding and survival.
